# Efficacy and safety of electroacupuncture for secondary sleep disorders: A meta-analysis and systematic review

**DOI:** 10.1097/MD.0000000000034150

**Published:** 2023-06-30

**Authors:** Shiping Liu, Jie Liu, Jinfeng Su, Fuli Zhang

**Affiliations:** a School of Basic Medicine, Heilongjiang University of Traditional Chinese Medicine: Heilongjiang University of Chinese Medicine, Harbin, China.

**Keywords:** electroacupuncture, insomnia, meta-analysis, secondary sleep disorder, systematic review

## Abstract

To explore the efficacy and safety of electroacupuncture (EA) for secondary insomnia through a meta-analysis and a systematic review.

**Methods:**

The CNKI, Wanfang, VIP database, Web of Science, EMBASE, PubMed, and Cochrane Library were retrieved. The retrieval date was February 28, 2023. Two independent reviewers conducted literature screening, data extraction, and risk of bias (ROB) assessment. The revised Cochrane ROB tool was used to assess the ROB in included studies. Data analysis was performed using RevMan 5.4 software and Stata 15.0.

**Results:**

Thirteen randomized controlled studies were included, involving 820 patients, including 414 patients in EA group and 406 patients in the control group. Compared with the control group, EA could improve secondary insomnia overall responses (relative risk = 3.90, 95% confidence interval [CI] [1.87, 8.13], *P* < .001), reduce Pittsburgh Sleep Quality Index score (mean difference [MD] = −2.26, 95% CI [−4.14, −0.37], *P* = .02), reduce Athens Insomnia Scale score (MD = −0.57, 95% CI [−2.70, 1.56], *P* = .60), prolonged total sleep time (MD = 2.63, 95% CI [−0.59, 5.86], *P* = .11), and not increase adverse events (relative risk = 0.50, 95% CI [0.18, 1.44], *P* = .20).

**Conclusion:**

EA may be a promising treatment for secondary sleep disorders; however, more high-quality studies are needed to confirm our findings.

## 1. Introduction

The emergence of secondary insomnia is hypothesized to be caused by interactions between predisposing variables and predisposing events. Secondary insomnia is a complex sleep condition. It is characterized by trouble falling asleep, early rising, light sleep, easy awakening, etc.^[1,2]^ Secondary insomnia is a complex sleep condition. It is characterized by trouble falling asleep, early rising, light sleep, easy awakening, etc.^[3]^ The short-term consequences of the disease comprise mental health issues, heightened reactions to stress, performance and cognitive deficiencies, physical discomfort, and a decreased standard of life.^[4]^ Along with illnesses such as dyslipidemia, hypertension, cardiovascular disease, type 2 diabetes, metabolic syndrome, and colorectal cancer, sleep disorders can also have long-term negative health effects.^[5]^Improved hormone management, immunity, reproductive health, mental health, cognition, and cardiovascular health are all benefits of getting enough sleep.^[6]^ About 10% of adults suffer from insomnia, and another 20% occasionally experience symptoms. Women, the elderly, and people with socioeconomic difficulties are more likely to suffer from insomnia. Therefore, it is important to address insomnia as a significant public health issue.^[7]^ A great deal of secondary sleep disturbances are brought through disorders which includes cancer, stroke, premenopausal syndrome, depression, and so on.The risk of stroke recurrence and mortality can be decreased by treating sleep problems.^[8]^ 25 to 59% of cancer patients are experiencing severe sleep problems, which is twice as common as in the general population.^[9]^ Patients with breast cancer are particularly prone to it.^[10]^ Additionally, sleep disorders can worsen cancer, lower quality of life, and impede recovery. Radiotherapy, chemotherapy, hormone therapy, and other cancer therapies are significant contributors to sleep problems.^[10]^ Medication and cognitive behavioral therapy are the 2 most common treatments for insomnia. Sedative-hypnotic medications are currently the mainstay of the therapy for insomnia. Nonbenzodiazepines and melatonin receptor agonists, such as Zolpidem, Zopiclone, Zaleplon, and others, are preferred in the diagnosis and treatment guidelines for insomnia because of their short half-lives, low next-day residual effect, effective treatment, and lack of serious adverse events.^[11,12]^ Long-term use of these medicines, however, can still have an adverse effect on motor, executive, and neurocognitive skills the following day.^[13]^ The sustained use of such medicines may also lessen the hypnotic effect.^[14]^ Important adverse effects of benzodiazepines have been noted, including dependency withdrawal syndrome, drug habit, memory issues, falls in the elderly, and daytime drowsiness.^[15]^

Moreover, for patients with sleep disorders with primary disease, the potential impact of multiple drug use can be significant. Electroacupuncture (EA) is a combination of acupuncture and electrical stimulation. EA therapy is based on the theory of meridians and collaterals in traditional Chinese medicine. Stimulate the points to achieve the effect of curing the disease. Insomnia is caused by an imbalance in the nervous system of the brain. EA can adjust the nervous system and improve its stress state.^[16]^ Studies have also found that acupuncture can regulate 1L-1β and brain-derived nerve growth factors to improve sleep quality.^[17]^ Animal experiments have shown that EA can improve insomnia by regulating the expression of adenosine 5’-monophosphate-activated protein kinase in the paraventricular nucleus of the hypothalamus and the content of Ac-Coa and Na^+^- K^+^- ATPase, thus regulating the energy metabolism in the paraventricular nucleus of the hypothalamus.^[18]^

There are disputes over the efficacy and safety of EA for secondary sleep disorders, so we hope to solve the above disputes through this study and improve a new choice for the clinical treatment of secondary sleep disorders. Therefore, to support the efficacy and safety of EA in the treatment of sleep disorders, a comprehensive review of the efficacy of EA in the treatment of sleep disorders at different stages needs to be conducted using the latest RCTS-updated data. Pittsburgh Sleep Quality Index (PSQI), Athens Insomnia Scale (AIS), Insomnia Severity Index (ISI), and total sleep time (TST) are important quantitative indicators to evaluate sleep quality and provide a reliable basis for the embodiment of clinical efficacy.

## 2. Methods

The protocol has been registered in the International Prospective Register of Systematic Reviews database (PROSPERO: CRD42023409120) and has no amendments.

### 2.1. Retrieval strategy

A systematic search of the literature before April 2022 was performed using databases (CNKI, Wanfang, VIP database, Web of Science, EMBASE, PubMed, Cochrane Library, etc.) to collect randomized controlled trials (RCTs) on EA intervention in patients with insomnia with search terms such as “electroacupuncture,” “sleep initiation and maintenance disorders,” “insomnia,” and “randomized controlled trial, RCT” (File 1). The systematic review followed the Preferred Reporting Items for Systematic Reviews and Meta-Analysis guidelines. In addition, references to the included studies were traced to obtain and supplement relevant references.Two independent reviewers conducted literature screening, data extraction, and risk of bias (ROB) assessment.

**File 1.**
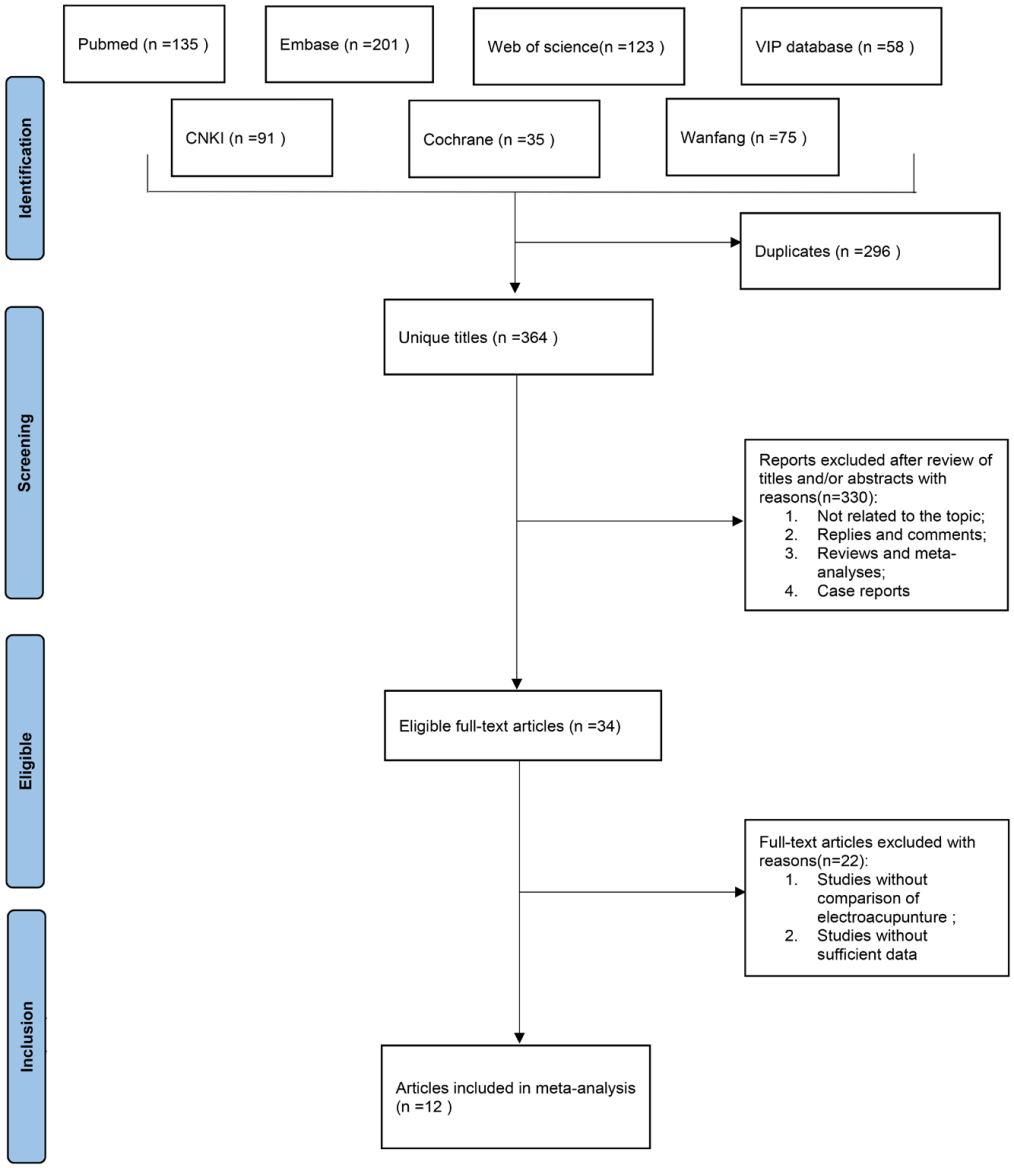
Process of study selection following the Preferred Reporting Items for Systematic Reviews and Meta-Analyses (PRISMA).

### 2.2. Inclusion and exclusion criteria

Studies were included if the following criteria were met: the study was an RCT; the target population was individuals with secondary sleep disorder; type of intervention:an experimental group included in the form of EA; EA was compared with a control group exposed to any or no intervention; PSQI score, AIS, TST, and ISI score, were evaluated as the outcomes and all included outcome indicators were assessed at the beginning and the end of the intervention; The exclusion criteria were as follows: non-RCT studies; animal studies, case reports, conference abstracts, and letters to the editor; insufficient data or irrelated outcomes.

### 2.3. Study Selection

Two independent assessors screened the literature and extracted data. The screening process involves reading titles and abstracts, as well as the full text of the literature, to find out what is easy to judge. The articles that need further evaluation will be downloaded in full. The inclusion and exclusion criteria were strictly followed during the screening process, and the observation indicators of the 2 groups of studies were extracted and cross-checked for consistency. Data extraction included the first author, year of publication, registration number, primary disease, sample size, and follow-up.If there was disagreement, an agreement was reached after a third reviewer evaluated the article.

### 2.4. ROB evaluation

Two review authors independently assessed the quality of the studies included in the analysis. To assess the quality of these studies, researchers used the bias analysis tool in the Cochrane Manual of Systematic Reviews of Interventions 5.1.0.19. The tool assessed studies based on 7 criteria, including random sequence generation, allocation concealment, blinding of implements and participants, blinding of outcome assessors, completeness of data results, selective reporting of study results, and other sources of bias. Each study assessed 7 areas to produce a comprehensive quality assessment. Methodological quality assessment tables, ROB plots, and summary ROB charts were then created from these assessments.

The quality of the evidence was generally good. Four of the 12 trials were rated as having a high overall ROB, 7 were rated as having some concern, and one was rated as having a low risk. Blind-related bias contributed the most to the high ROB, which may be attributed primarily to the nature of EA, which excluded blinding for patient and clinician treatment. Work to reduce patient and evaluator bias.

### 2.5. Data extraction and analysis

Details of the retrieved articles are summarized in Table 1. Data regarding the study characteristics (first author; year of publication; country; trial design; mean age; sample size; intervention, outcome measures, and adverse events) of each article were extracted. The passive intervention was defined as the blank control in the control group, whereas the acupuncture or regular treatment in the experimental group was defined as the active intervention. Any conflicts or ambiguities in the reporting methods or results during data extraction were discussed with a third reviewer and resolved by consensus.

**Table 1:** Characteristics of randomized controlled trials included in the meta-analysis.

			Participant characteristics		Intervention protocol			
References	Country	Study design	Age, mean (SD) e.g.; SG	N (e.g./SG)	Intervention	Control	Outcome	Adverse effects
Zhou 2022	China	RCT	66 (5); 68 (6)	60 (30/30)	Electroacupuncture	Regular treatment	PSQI, AIS	NR
Yu 2022	China	RCT	49.7 (3.2); 48.8 (3.4)	60 (30/30)	Electroacupuncture	Sham acupuncture	PSQI	NR
Yin 2022	China	RCT	50.9 (14.0); 50.5 (14.0)	180 (90/90)	Electroacupuncture	Sham acupuncture	PSQI	Hematoma and local pain (n = 7)
Lee 2022	Korea	RCT		14 (8/6)	Electroacupuncture	Sham acupuncture	PSQI, ISI	NR
Liu 2021	China	RCT	62. 83 (7. 48); 62. 60 (7. 04)	60 (30/30)	Electroacupuncture	Acupuncture	PSQI, AIS	NR
Yin 2020	China	RCT	47.30 (14.89); 49.80 (15.13)	60 (30/30)	Electroacupuncture	Sham acupuncture	PSQI, TST	Numbness and pain (n = 3)
Li 2020	China	RCT	52.12 (4.19); 53.07 (3.81)	84 (42/42)	Electroacupuncture	Sham acupuncture	PSQI, ISI, TST	Bleeding (n = 1) pain (n = 1)
Zhao 2019	China	RCT	52.1 (4.1); 51.9 (3.9)	66 (33/33)	Electroacupuncture	Regular treatment	PSQI	Dizziness (n = 1)
Jiang 2019	China	RCT	46.05 (16.15); 46.10 (17.22)	70 (35/35)	Electroacupuncture	Regular treatment	PSQI	NR
Chen 2011	China	RCT	48 ± 6; 48 ± 7	70 (38/32)	Electroacupuncture	Regular treatment	AIS	NR
Yeung 2011	China	RCT	47.5 (8.5); 50.1 (9.1)	52 (26/26)	Electroacupuncture	Sham acupuncture	PSQI, ISI,TST	Headache (n = 2) Dizziness (n = 1)
Mao 2014	America	RCT	57.5 (10.1); 60.9 (6.5)	44 (22/22)	Electroacupuncture	Sham acupuncture	PSQI	NR

AIS = Athens Insomnia Scale, ISI = Insomnia Severity Index, PSQI = Pittsburgh Sleep Quality Index, RCT = randomized controlled trial, TST = total sleep time.

The data extracted from the study were entered into the statistical software Revman 5.4 and Stata 15.0 (StataCorp, College Station, TX) for analysis. Heterogeneity was tested with *I*^2^ values or *Q* statistics. The *I*^2^ values were 0%, 25%, 50%, and 75%, respectively, with no heterogeneity, low heterogeneity, moderate heterogeneity, and high heterogeneity. When *I*^2^ is <50%, sensitivity analyses are performed to explore potential sources of heterogeneity. Where heterogeneity was <50%, a fixed-effects model was used. In addition, funnel plots were used to assess publication bias.

## 3. Result

### 3.1. Literature screening

A total of 660 articles were preliminarily retrieved; 581 articles were acquired after removing duplicates; 34 articles were obtained by checking the titles and abstracts of the articles; and 12 articles^[2,16,19–28]^ were finally included in the analysis by reading the full text. Animal experiments, combination therapy, etc are excluded from the literature.

### 3.2. The basic characteristics table of the included literature

A total of 12 randomized controlled studies were included, involving a total of 820 patients, including 414 patients in the EA group (E) and 406 patients in the control group (C). The specific features of the paper are shown in Table 1.

### 3.3. ROB assessment

**Figure 1.**
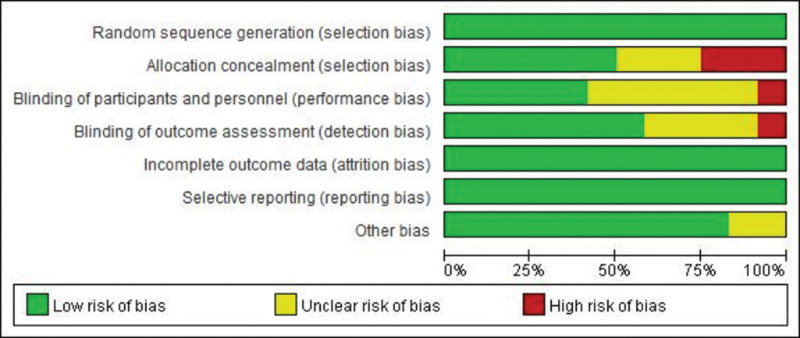
Assessment of risk of bias with selected studies.

The 12 articles included in this study all explained the method of randomization and the blind method used, and 7 articles explained the blind method used for outcome evaluators. The bias risk of the articles is shown in Figure 1.

### 3.4. Meta-analysis of overall responses

**Figure 2.**
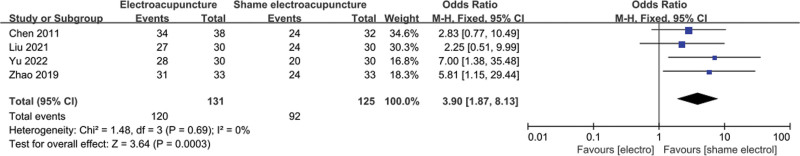
Forest plot for overall response.

The overall response was reported in 4 studies, including 131 patients in the EA group and 125 patients in the control group. The heterogeneity test (*I*^2^ = 0%, *P* = .69), the random effects model, was used for data analysis. The analysis results (relative risk = 3.90, 95% confidence interval [CI] [1.87, 8.13], *P* < .001) suggested that the overall response was improved by EA compared with the control group, as shown in Figure 2.

### 3.5. Meta-analysis of change in PSQI score

**Figure 3.**
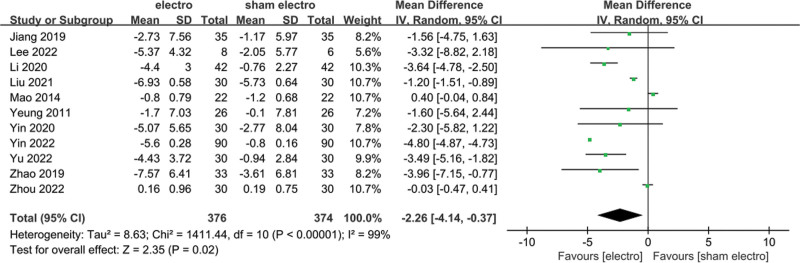
Forest plot for PSQI score. PSQI = Pittsburgh Sleep Quality Index.

**Figure 4.**
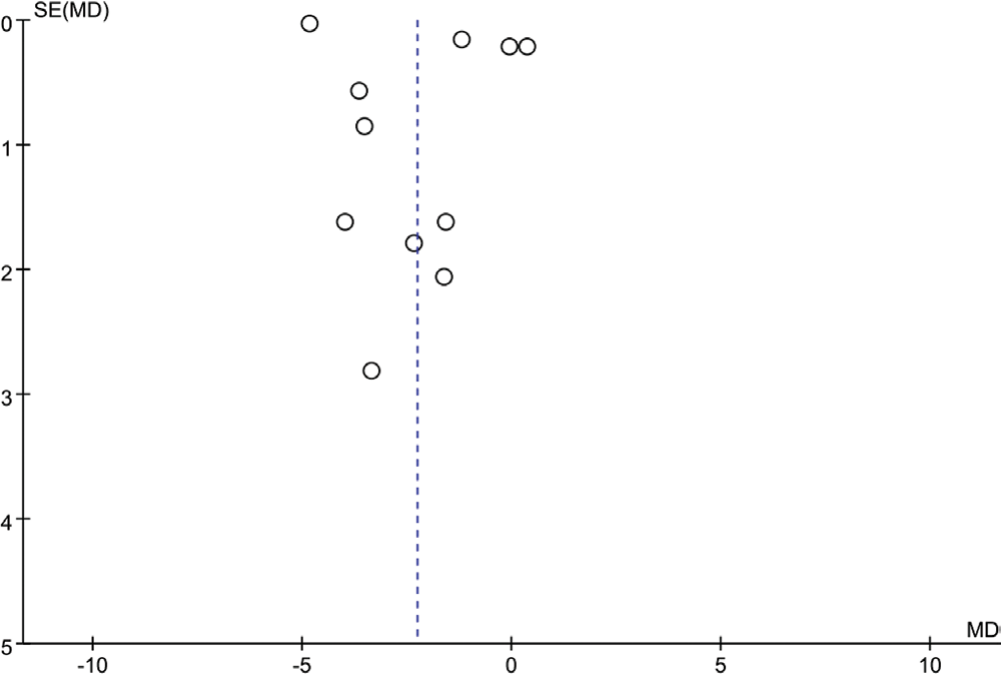
Sensitivity analysis for PSQI score. PSQI = Pittsburgh Sleep Quality Index.

Eleven studies mentioned changes in PSQI scores: 376 patients in the EA group and 374 patients in the control group. A heterogeneity test (*I*^2^ = 99%, *P* < .01) and a random effects model were used for data analysis. The analysis results (mean difference [MD] = −2.26, 95% CI [−4.14, −0.37], *P* = .02) suggested that PSQI scores could be reduced by EA compared with the control group, as shown in Figure 3. When *I*^2^ = 50%, the sensitivity analysis of this index was carried out, and the analysis results showed that the sensitivity was small and the result was relatively stable, as shown in Figure 4.

### 3.6. Meta-analysis of change in AIS score

**Figure 5.**
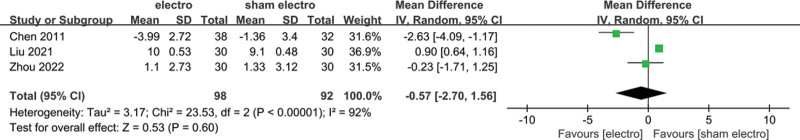
Forest plot for AIS score. AIS = Athens Insomnia Scale.

**Figure 6.**
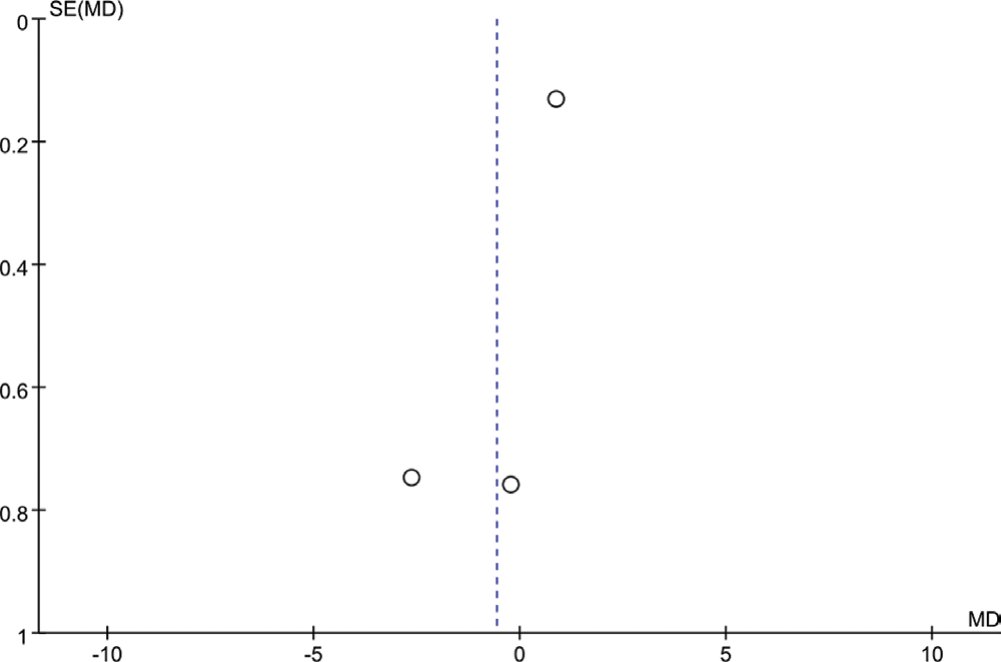
Sensitivity analysis for AIS score. AIS = Athens Insomnia Scale.

Three studies mentioned changes in AIS scores: 98 patients in the EA group and 92 patients in the control group. Heterogeneity was tested (*I*^2^ = 92%, *P* < .01), and a random-effects model was used for data analysis. The results (MD = −0.57, 95% CI [−2.70 to 1.56], *P* = .60) suggested that EA reduced AIS scores compared with controls, as shown in Figure 5. When As *I*^2^ was 50%, the sensitivity analysis of this index was carried out, and the analysis results showed that the sensitivity was small and the result was relatively stable, as shown in Figure 6.

### 3.7. Meta-analysis of change in ISI score

**Figure 7.**
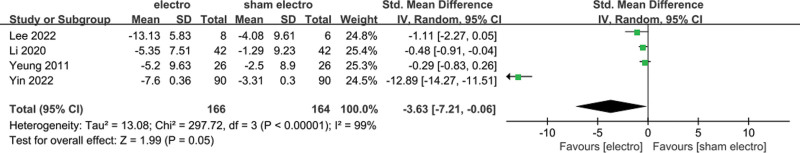
Forest plot for ISI score. ISI = Insomnia Severity Index.

**Figure 8.**
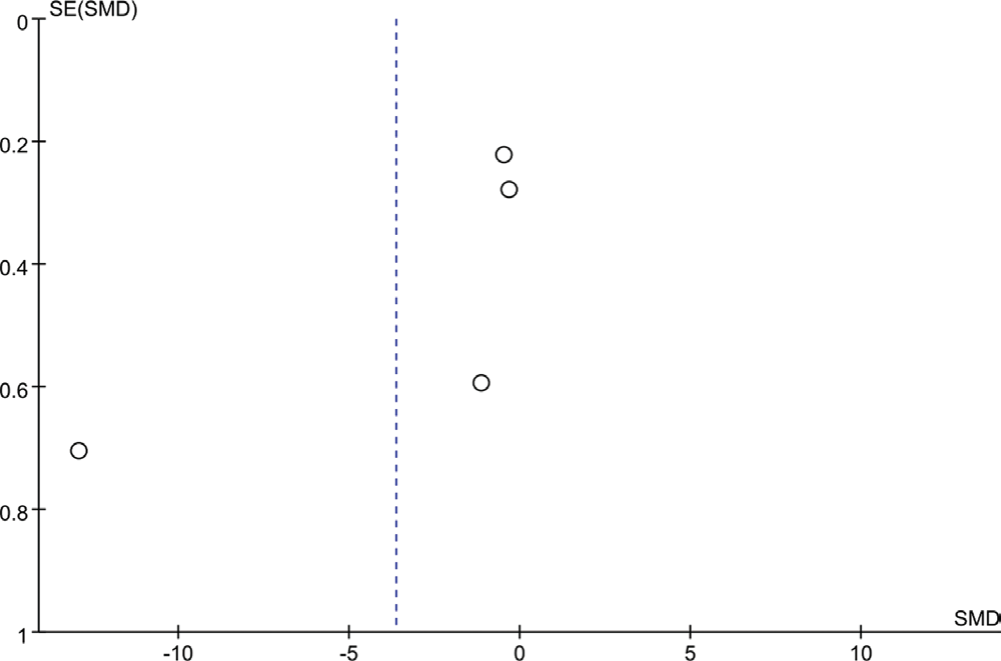
Sensitivity analysis for ISI score. ISI = Insomnia Severity Index.

Four studies mentioned changes in ISI scores: 166 patients in the EA group and 164 patients in the control group. Heterogeneity was tested (*I*^2^ = 99%, *P* < .001), and a random-effects model was used for data analysis. The results (MD = −3.63, 95% CI [−7.21 to −0.06], *P* = .05) suggested that EA reduced ISI scores compared with controls, as shown in Figure 7. When As *I*^2^ was 50%, the sensitivity analysis of this index was carried out, and the analysis results showed that the sensitivity was small and the result was relatively stable, as shown in Figure 8.

### 3.8. Meta-analysis of the change in TST

**Figure 9.**
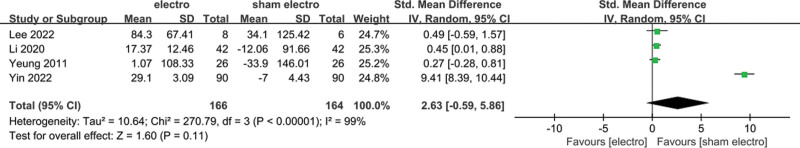
Forest plot for TST. TST = total sleep time.

**Figure 10.**
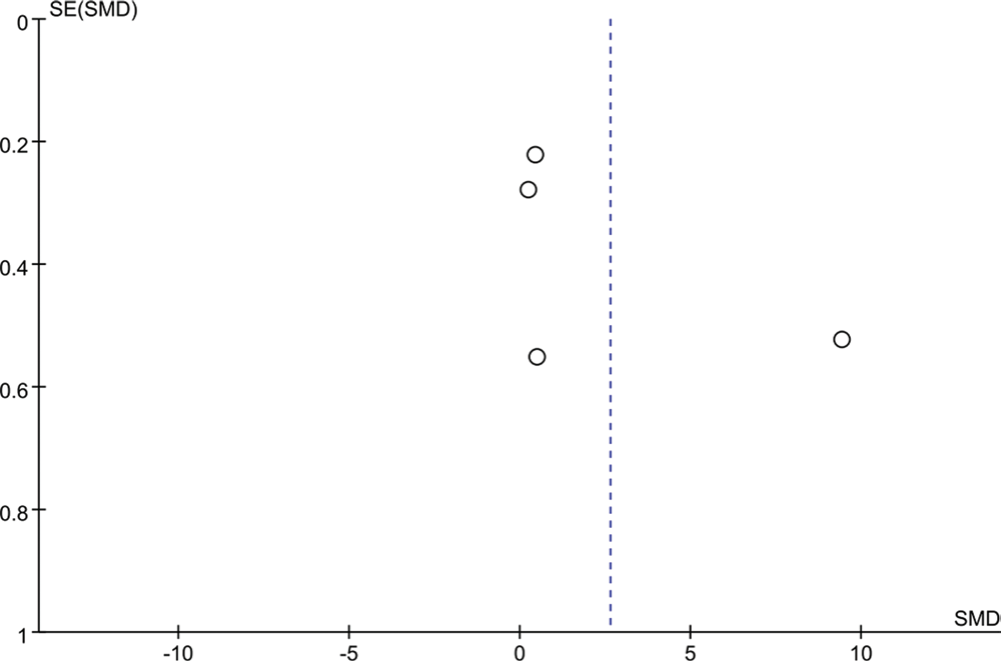
Sensitivity analysis for TST. TST = total sleep time.

Four studies mentioned changes in TST, involving 166 patients in the EA group and 164 patients in the control group. The heterogeneity test (*I*^2^ = 99%, *P* < .001) and the random effects model were used for data analysis. Results (MD = 2.63; 95% CI [−0.59 to 5.86]; *P* = .11) showed that TST was prolonged by EA compared with the control group, as shown in Figure 9. When As *I*^2^ was 50%, the sensitivity analysis of this index was carried out, and the analysis results showed that the sensitivity was small and the result was relatively stable, as shown in Figure 10.

### 3.9. Meta-analysis of adverse events

**Figure 11.**
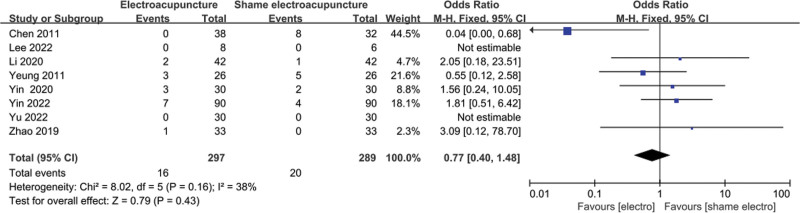
Forest plot for adverse events.

Adverse events were mentioned in 8 studies, including 297 patients in the EA group and 289 patients in the control group. The heterogeneity test (*I*^2^ = 38%, *P* = .16) and the random effects model were used for data analysis. The analysis results (relative risk = 0.77, 95% CI [0.40, 1.48], *P* = .43) suggested that, compared with the control group, there was no increase in adverse events in the EA group (Fig. 11).

## 4. Discussion

A total of 12 trials with 820 participants were included, and the data were pooled to perform a systematic review and meta-analysis. Most of the trials were based in mainland China, while some were conducted in America and Hong Kong. Treatment points vary from test to test, mainly the basic Neiguan, Shenmen, and other acupoints. The control group received conventional medication. Through data analysis, it is concluded that EA has a good therapeutic effect on secondary insomnia, can effectively reduce PSQI, ISI, and other scores, and is relatively low-cost.

The side effects of sleep disorder medications frequently include drug treatment and dependence, addiction potential, and severe responses.^[29]^ A form of acupuncture called EA uses electrodes to deliver a pulsed current. Although it’s still debatable, EA has been proven to be more effective than acupuncture in treating some diseases. Acupuncture offers anti-inflammatory, anti-anxiety, antidepressive, gut flora improvement, and immune function regulation therapeutic benefits.^[30]^ Compared with primary insomnia, the effect of electricity on secondary insomnia was more significant than in the control group.^[28,31]^ Studies have indicated that in people with insomnia, inflammatory cytokines including IL-6 and TNF-α, as well as the gut flora, are more disrupted. Additionally, the microbiome structure varies depending on the stage of insomnia.^[31]^ EA can enhance endorphins, which aid in sleep promotion, and have a long-lasting impact on autonomic nerve activity.^[32,33]^ Multi-target activity may be the potential mechanism of EA.^[34]^

The occurrence of secondary sleep disorders is closely related to the primary disease. Take the primary disease included in the literature as an example. Patients with depression have more REM sleep or a shorter REM latency period, which means they may sleep less.^[35]^ In a similar vein, depression might become more severe due to insomnia.^[36]^ Endocrine hormones and inflammation are both important factors in this process.^[35]^ For instance, serotonin has been connected to both the start of REM sleep state and the emergence of depression. Sleep deprivation alters the microbiota, which can impair immunological and neurological system function.^[37]^ The test results suggest that EA can not only improve sleep quality but also improve mental states in patients with depression or menopause.^[23]^ Sleep disorders affect 25 to 59 percent of cancer patients. Breast cancer and gynecological cancer patients had the highest rates of insomnia.^[38]^ Insomnia can also be triggered by the gender and location of the tumor.^[39]^ On the other hand, insomnia can result in exhaustion, worry, and a deterioration of the body’s immunity.^[40]^ One of the main causes for behavioral cognitive therapy’s difficulty in treating these individuals is the low compliance and nonresponse of some patients.^[41]^ Another crucial aspect to think about is how sedatives and cancer medications interact with one another. During menopause, insomnia is roughly twice as common.^[42]^ According to studies, menopause-related changes in estrogen, neurotransmitter imbalances, and psychological factors can all result in poorer sleep quality and, ultimately, sleep problems. Insomnia might make symptoms of menopause worse.^[43]^ Both perimenopausal depression and insomnia are bidirectional intensifications with high prevalence rates.^[42]^ A decreased estradiol level and decreased hypothalamus sensitivity to estrogen are all precursors of sleep disorders.^[44]^ According to clinical trials, acupuncture has a better than expected medium-term efficacy in treating secondary insomnia.^[45]^

It is unclear exactly how EA works to treat sleep disturbances. Experts predict that nondrug therapy for the treatment of sleep disorders will gradually play a major role in enhancing sleep quality and reducing sleep symptoms as more is learned about the disease’s pathophysiology. This essay concludes that EA can successfully treat sleep problems and insomnia. The following are the main mechanisms of action: First of all, increasing numbers of research have revealed a strong correlation between sleep disorders and gut flora imbalance, and that controlling this relationship is key to controlling insomnia. Intestinal flora has a significant impact on sleep through the microbiome-gut-brain axis.^[46]^ Short sleep times will cause a physiological stress response in the human body, which will destroy the balance and stability of intestinal flora and induce metabolic diseases.^[47]^ Secondly, EA stimulates the vagus nerve, parasympathetic nerve, and other neurotransmitters acting on the sleep center, such as melatonin, adrenaline, and so on.^[48]^ Melatonin actively regulates sleep, shortening sleep duration and prolonging slow-wave sleep. Some scholars have found that electric acupuncture can stimulate the pineal gland to increase the secretion of melatonin, thus improving the quality of sleep.^[49–51]^ In addition, by directly affecting the autonomic nervous system, it can inhibit excessive arousal of the nervous system, but the specific mechanism is not clear. But it does affect some measures of autonomic nervous function, such as blood pressure, heart rate, neuro vasomotor activity, and pupil size. Or, it may be related to intense muscle contractions caused by acupuncture.^[50]^

EA can not only carry out repeated mechanical movements of acupuncture and improve the efficiency of acupuncture, but also objectively control the amount of stimulation and further improve the standardized operation of acupuncture, rather than the subjectivity of manual acupuncture.^[25]^ It is believed that EA can enhance local stimulation, promote the regular circulation of blood, and regulate the rhythm of nerve activity so that the effect of treatment can be sustained for a long time.^[26]^ The use of EA significantly improved sleep efficiency, increased TST and decreased the number of sleep awakenings. The difference in outcome indexes may be related to the choice of acupuncture points, the acupuncturist’s technique, needle selection, etc. However, in the included RCT literature, the selection of experienced acupuncturists was a necessary condition for the trial. Doctors with acupuncture experience of >5 years are generally selected for the operation.

## 5. Limitation

The stimulating current generated by EA is delivered to the acupoint through the inserted needle. This study is based on existing published research and is inevitably limited and influenced by factors beyond our control. As EA is a traditional treatment and a feature of traditional Chinese medicine, half of the included articles are in Chinese, which may lead to mild publication bias. The number of included articles is limited, so more clinical studies with more centers and large samples should be included in the future to ensure the research is extensive and low-risk. Among the included literature, 4 studies focused on sleep disorders during perimenopause, which resulted in all the patients participating in the study being female, which may have biased the analysis results. The acupuncturist’s technique is the key to the experiment. Each acupuncturist’s clinical level is different, so the effect of acupuncture will be slightly different. However, the experiment requires a sense of “de qi” as the condition for accurate acupuncture, which relatively reduces the inaccuracy caused by individual differences among acupuncturists.

## 6. Conclusion

According to the current study, EA improves the overall treatment effect of secondary sleep disorders, reduces PSQI and ASI scores, and prolongs TST without increasing adverse reactions. However, due to the limitations of the included literature collected in this study, we hope to have more high-quality, large-sample studies in future studies to support our findings.

## Author contributions

**Conceptualization:** Shiping Liu.

**Data curation:** Shiping Liu.

**Methodology:** Jinfeng Su.

**Project administration:** Jinfeng Su.

**Writing – original draft:** Shiping Liu, Jie Liu.

**Writing – review & editing:** Fuli Zhang.

## References

[1] Amin N Allebrandt KV van der Spek A. Genetic variants in RBFOX3 are associated with sleep latency. Eur J Hum Genet. 24:1488–95. 10.1038/ejhg.2016.31 PMC5027680 27142678

[2] Yu X Mou Y Sun X. Effects of electrotherapy on sleep and quality of life in perimenopausal insomnia patients with liver-kidney Yin deficiency. Chin Med Inf. 2022;33:6:69–73.

[3] Lee S Oh JW Park KM. Digital cognitive behavioral therapy for insomnia on depression and anxiety: a systematic review and meta-analysis. NPJ Digit Med. 2023;6:52. 36966184 10.1038/s41746-023-00800-3 PMC10039857

[4] Al Lihabi A. A literature review of sleep problems and neurodevelopment disorders. Front Psychiatry. 2023;14:1122344. 36911135 10.3389/fpsyt.2023.1122344 PMC9995546

[5] Abbott SM Videnovic A. Chronic sleep disturbance and neural injury: links to neurodegenerative disease. Nat Sci Sleep. 2016;8:55–61. 26869817 10.2147/NSS.S78947 PMC4734786

[6] Baranwal N Yu PK Siegel NS. Sleep physiology, pathophysiology, and sleep hygiene. Prog Cardiovasc Dis. 2023:S0033-0620(23)00011-7. 10.1016/j.pcad.2023.02.005 36841492

[7] Morin CM Jarrin DC. Epidemiology of insomnia: prevalence, course, risk factors, and public health burden. Sleep Med Clin. 2022;17:173–91. 35659072 10.1016/j.jsmc.2022.03.003

[8] Hale E Gottlieb E Usseglio J. Post-stroke sleep disturbance and recurrent cardiovascular and cerebrovascular events: a systematic review and meta-analysis. Sleep Med. 2023;104:29–41. 36889030 10.1016/j.sleep.2023.02.019 PMC10098455

[9] Savard J Morin CM. Insomnia in the context of cancer: a review of a neglected problem. J Clin Oncol. 2001;19:895–908. 11157043 10.1200/JCO.2001.19.3.895

[10] Fiorentino L Ancoli-Israel S. Sleep dysfunction in patients with cancer. Curr Treat Options Neurol. 2007;9:337–46. 17716597 PMC2951736

[11] Zhang P Li YP. Guidelines for the diagnosis and treatment of adult insomnia in China. Chin J Neur. 2018;5:324–35.

[12] He QN Wang XD. Research progress of drug therapy for chronic insomnia. Chin J Clin Pharmacol. 2018;15:1932–6.

[13] Sun YK Shi L Chen SQ. Effects of sedative-hypnotic drug therapy on cognitive function in patients with insomnia. Chin J Nerv Ment Dis. 2017;11:701–4.

[14] Zhao FY Duan YR Yan HX. Clinical evaluation of moxibustion combined with Taijiquan and Jacobson progressive muscle relaxation training on exercise-induced insomnia. J Shenyang Sport Univ. 2016;5:75–80.

[15] Gunja N. The clinical and forensic toxicology of Z-drugs. J Med Toxicol. 2013;9:155–62. 23404347 10.1007/s13181-013-0292-0 PMC3657020

[16] Chen X Xu K Qin X. Treatment of perimenopausal insomnia by electroacupuncture. Shanghai J Acupunct Moxibustion. 2011;30:366–7.

[17] Qiao L Gao Q Tan L. Effects of electroacupuncture on interleukin-1β, brain-derived nerve growth factor and other sleep-related factors in serum of sleeping rats. Acupunct Res. 2018;43:651–6.

[18] Zhu Y Yang C He L. Effects of electroacupuncture on energy metabolism of paraventricular nucleus of hypothalamus in insomnia rats. J Acupunct Res. 2019;44:170–5. 10.13702/j.1000-0607.170996 30945498

[19] Zhou Y Zhou M. Electric acupuncture preconditioning the effects of sleep quality in patients with laparoscopic gastric cancer radical surgery. J Shanghai Acupunct Mag. 2022;9:1181–4.

[20] Yin X Li W Wu H. Efficacy of electroacupuncture on treating depression-related insomnia: a randomized controlled trial. Nat Sci Sleep. 2020;12:497–508. 32765146 10.2147/NSS.S253320 PMC7382580

[21] Lee B Kim BK Kim M. Electroacupuncture for treating cancer-related insomnia: a multicenter, assessor-blinded, randomized controlled, pilot clinical trial. BMC Complement Med Ther. 2022;22:77. 35303841 10.1186/s12906-022-03561-w PMC8932204

[22] Liu Y Chen C. Acusector door god, the acupuncture point to treat insomnia after stroke clinical observation. J Chin Med Inf. 2021;38:68–71.

[23] Yin X Li W Wu H. Efficacy of electroacupuncture on treating depression-related insomnia: a randomized controlled trial. Nat Sci Sleep. 2020;12:497–508. 32765146 10.2147/NSS.S253320 PMC7382580

[24] Li S Wang Z Wu H. Electroacupuncture versus sham acupuncture for perimenopausal insomnia: a randomized controlled clinical trial. Nat Sci Sleep. 2020;12:1201–13. 33376432 10.2147/NSS.S282315 PMC7764880

[25] Zhao FY Yan HX. Efficacy and safety of electroacupuncture for perimenopausal insomnia: a randomized controlled trial. J Acupunct Tuina Sci. 2019;17:188–95.

[26] Jiang Y Wang L Li Y. Clinical study on treatment of sudden deafness with tinnitus, anxiety and sleep disorders by electroacupuncture. Clin J Acupunct Moxibustion. 2019;35:38–40.

[27] Mao JJ Farrar JT Bruner D. Electroacupuncture for fatigue, sleep, and psychological distress in breast cancer patients with aromatase inhibitor-related arthralgia: a randomized trial. Cancer. 2014;120:3744–51. 25077452 10.1002/cncr.28917 PMC4239308

[28] Yeung WF Chung KF Zhang SP. Electroacupuncture for primary insomnia: a randomized controlled trial. Sleep. 2009;32:1039–47. 19725255 10.1093/sleep/32.8.1039 PMC2717194

[29] National Institutes of Health State of the Science Conference Statement on manifestations and management of chronic insomnia in adults, June 13–15, 2005. Sleep. 2005;28:1049–57. 16268373 10.1093/sleep/28.9.1049

[30] Zhang B Shi H Cao S. Revealing the magic of acupuncture based on biological mechanisms: a literature review. Biosci Trends. 2022;16:73–90. 35153276 10.5582/bst.2022.01039

[31] Li Y Zhang B Zhou Y. Gut microbiota changes and their relationship with inflammation in patients with acute and chronic insomnia. Nat Sci Sleep. 2020;12:895–905. 33177907 10.2147/NSS.S271927 PMC7652227

[32] Huang W Kutner N Bliwise DL. Autonomic activation in insomnia: the case for acupuncture. J Clin Sleep Med. 2011;7:95–102. 21344045 PMC3041619

[33] Cheng CH Yi PL Lin JG. Endogenous opiates in the nucleus tractus solitarius mediate electroacupuncture-induced sleep activities in rats. Evid Based Complement Alternat Med. 2009. 10.1093/ecam/nep132 PMC3094708 19729491

[34] Zhang Z Cai X Liang Y. Electroacupuncture as a rapid-onset and safer complementary therapy for depression: a systematic review and meta-analysis. Front Psychiatry. 2023;13:1012606. 36684018 10.3389/fpsyt.2022.1012606 PMC9853905

[35] Edge LC. The role of emotional brain processing during sleep in depression. J Psychiatr Ment Health Nurs. 2010;17:857–61. 21078000 10.1111/j.1365-2850.2010.01598.x

[36] Chung KH Li CY Kuo SY. Risk of psychiatric disorders in patients with chronic insomnia and sedative-hypnotic prescription: a nationwide population-based follow-up study. J Clin Sleep Med. 2015;11:543–51. 25766696 10.5664/jcsm.4700 PMC4410928

[37] Dinan TG Cryan JF. Mood by microbe: towards clinical translation. Genome Med. 2016;8:36. 27048547 10.1186/s13073-016-0292-1 PMC4822287

[38] Savard J Villa J Ivers H. Prevalence, natural course, and risk factors of insomnia comorbid with cancer over a 2-month period. J Clin Oncol. 2009;27:5233–9. 19738124 10.1200/JCO.2008.21.6333

[39] Chan Y Irish JC Wood SJ. Smoking cessation in patients diagnosed with head and neck cancer. J Otolaryngol. 2004;33:75–81. 15518093 10.2310/7070.2004.00075

[40] Yao JL Tian JH. On the treatment of tumor related insomnia with the combination of form and spirit. Shanxi Trad Chin Med. 2020;41:213–6.

[41] Matthews EE Schmiege SJ Cook PF. Adherence to cognitive behavioral therapy for insomnia (CBTI) among women following primary breast cancer treatment: a pilot study. Behav Sleep Med. 2012;10:217–29. 22742439 10.1080/15402002.2012.666220

[42] Caruso D Masci I Cipollone G. Insomnia and depressive symptoms during the menopausal transition: theoretical and therapeutic implications of a self-reinforcing feedback loop. Maturitas. 2019;123:78–81. 31027682 10.1016/j.maturitas.2019.02.007

[43] Zhao M Sun M Zhao R. Effects of exercise on sleep in perimenopausal women: a meta-analysis of randomized controlled trials. Explore (NY). 2023;S1550-8307(23)00030-7. 10.1016/j.explore.2023.02.001 36781319

[44] Parry BL Fernando Martnez L Maurer EL. Sleep, rhythms and women’s mood. Part II. Menopause. Sleep Med Rev. 2006;10:197–208. 16618548 10.1016/j.smrv.2005.09.004

[45] Zhao FY Zheng Z Fu QQ. Acupuncture for comorbid depression and insomnia in perimenopause: a feasibility patient-assessor-blinded, randomized, and sham-controlled clinical trial. Front Public Health. 2023;11:1120567. 36815166 10.3389/fpubh.2023.1120567 PMC9939459

[46] Li Y Hao Y Fan F. The role of microbiome in insomnia, circadian disturbance and depression. Front Psychiatry. 2018;9:669. 30568608 10.3389/fpsyt.2018.00669 PMC6290721

[47] Reynolds AC Paterson JL Ferguson SA. The shift work and health research agenda: considering changes in gut microbiota as a pathway linking shift work, sleep loss and circadian misalignment, and metabolic disease. Sleep Med Rev. 2017;34:3–9. 27568341 10.1016/j.smrv.2016.06.009

[48] Ou Y Lin D Ni X. Acupuncture and moxibustion in patients with cancer-related insomnia: a systematic review and network meta-analysis. Front Psychiatry. 2023;14:1108686. 36873228 10.3389/fpsyt.2023.1108686 PMC9979218

[49] Zheng X Wu X Guo X. Effects of acupuncture at different acupuncture points on Melatonin content in pineal gland of insomnia rats. Acupunct Res. 2018;43:360–4.

[50] Xie Z Chen F Li W. A review of sleep disorders and melatonin. Neurologic Res. 2017;39:559–65. 10.1080/01616412.2017.1315864 28460563

[51] Pan X Zhang L Yang Y. Therapeutic effect of electroacupuncture in treating moderate and severe insomnia. J Cardiovasc Dis Integr Tradit West Med. 2016;4:51–52, 54.

[52] Huang W Kutner N Bliwise DL. Autonomic activation in insomnia: the case for acupuncture. J Clin Sleep Med. 2011;7:95–102. 21344045 PMC3041619

